# Repeated endo-tracheal tube disconnection generates pulmonary edema in a model of volume overload: an experimental study

**DOI:** 10.1186/s13054-022-03924-2

**Published:** 2022-02-18

**Authors:** Bhushan H. Katira, Doreen Engelberts, Sheena Bouch, Jordan Fliss, Luca Bastia, Kohei Osada, Kim A. Connelly, Marcelo B. P. Amato, Niall D. Ferguson, Wolfgang M. Kuebler, Brian P. Kavanagh, Laurent J. Brochard, Martin Post

**Affiliations:** 1 Translational Medicine Program, Hospital for Sick Children, Peter Gilgan Centre for Research and Learning, 686 Bay St., 9th Floor, Toronto, ON M5G 0A4 Canada; 2 The Institute of Medical Sciences, University of Toronto, Toronto, ON Canada; 3 Division of Critical Care Medicine, Department of Paediatrics, Washington University School of Medicine, St Louis, MO USA; 4 Department of Medical Biophysics, University of Toronto, Toronto, ON Canada; 5 School of Medicine and Surgery, University of Milan-Bicocca, Monza, Italy; 6 Keenan Research Centre for Biomedical Sciences, Li Ka Shing Knowledge Institute, St Michael’s Hospital, Toronto, Canada; 7 Laboratório de Pneumologia LIM–09, Disciplina de Pneumologia, Instituto do Coração (Incor) Hospital das Clínicas da Faculdade de Medicina da Universidade de São Paulo, São Paulo, Brazil; 8 Division of Respirology, Department of Medicine, University Health Network and Sinai Health Systems, Toronto, ON Canada; 9 Interdepartmental Division of Critical Care Medicine, University of Toronto, Toronto, ON Canada; 10 Institute of Physiology, Charité – Universitätsmedizin Berlin, Berlin, Germany; 11 Departments of Critical Care Medicine and Anaesthesiology, Hospital for Sick Children, University of Toronto, Toronto, ON Canada

**Keywords:** Lung deflation, Pulmonary oedema, Pulmonary vascular resistance

## Abstract

**Background:**

An abrupt lung deflation in rodents results in lung injury through vascular mechanisms. Ventilator disconnections during endo-tracheal suctioning in humans often cause cardio-respiratory instability. Whether repeated disconnections or lung deflations cause lung injury or oedema is not known and was tested here in a porcine large animal model.

**Methods:**

Yorkshire pigs (~ 12 weeks) were studied in three series. First, we compared PEEP abruptly deflated from 26 cmH_2_O or from PEEP 5 cmH_2_O to zero. Second, pigs were randomly crossed over to receive rapid versus gradual PEEP removal from 20 cmH_2_O. Third, pigs with relative volume overload, were ventilated with PEEP 15 cmH_2_O and randomized to repeated ETT disconnections (15 s every 15 min) or no disconnection for 3 h. Hemodynamics, pulmonary variables were monitored, and lung histology and bronchoalveolar lavage studied.

**Results:**

As compared to PEEP 5 cmH_2_O, abrupt deflation from PEEP 26 cmH_2_O increased PVR, lowered oxygenation, and increased lung wet-to-dry ratio. From PEEP 20 cmH_2_O, gradual versus abrupt deflation mitigated the changes in oxygenation and vascular resistance. From PEEP 15, repeated disconnections in presence of fluid loading led to reduced compliance, lower oxygenation, higher pulmonary artery pressure, higher lung wet-to-dry ratio, higher lung injury score and increased oedema on morphometry, compared to no disconnects.

**Conclusion:**

Single abrupt deflation from high PEEP, and repeated short deflations from moderate PEEP cause pulmonary oedema, impaired oxygenation, and increased PVR, in this large animal model, thus replicating our previous finding from rodents. Rapid deflation may thus be a clinically relevant cause of impaired lung function, which may be attenuated by gradual pressure release.

**Supplementary Information:**

The online version contains supplementary material available at 10.1186/s13054-022-03924-2.

## Background

Ventilator-induced lung injury (VILI) contributes to high mortality in acute respiratory distress syndrome (ARDS) [1–3]. Newer mechanisms of VILI are being recognised; recently, using an *in-vivo* rat model, we have identified a form of lung injury that is caused by an abrupt decrease in airway pressure following sustained inflation (i.e. acute lung deflation) [4]. Attention to acute lung deflation is important since deflation events resulting from endo-tracheal tube disconnects during suctioning or transport may occur several times a day in the care of critically ill ventilated patients. Such deflations result in altered respiratory mechanics [5] and reduced oxygenation, alveolar de-recruitment and reduced lung volume [6, 7].

In the rat model, this deflation injury, resulted from increased hemodynamic forces viz forward vascular surge and backward increased left ventricular filling pressure at the time of deflation, resulting in endothelial disruption, microvascular leak, pulmonary oedema, inflammation, acute cor-pulmonale and systemic hypotension [4]. Gradual deflation and lower systemic vascular pressures were protective against this injury. Deflation energetics have also been proposed as a mechanism of deflation injury [8]. Clinically, such deflations, can potentially contribute to altered respiratory function, and may add injury or oedema, thereby impacting outcomes [9–11].

Before extrapolating the findings of the rat model to humans, we conducted a series of experiments in a clinically relevant porcine model simulating acute deflation from abrupt airway pressure removal, as well as repeated deflations from endo-tracheal tube disconnects in a model of volume overload. We hypothesized that deflation injury occurs in large animal models (pigs), may be prevented by gradual deflation, and is equally evident upon frequent disconnections in the presence of relative fluid overload.

## Methods

All experiments were conducted according to the Canadian Animal Care guidelines and were approved by the Animal Care Committee at The Hospital for Sick Children, Toronto, Canada (animal use protocol # 58058).

### Animal preparation

27 healthy female Yorkshire pigs (36.5 ± 3.9 kg) were studied in 3 series of experiments. Intubation was done under sedation (Ketamine 12 mg∙kg^−1^) and anaesthesia (Pentobarbital 10 mg∙kg^−1^∙h^−1^), muscle paralysis (Rocuronium bolus 1 mg kg^−1^ followed by infusion at 0.02 mg∙kg^−1^∙h^−1^) and ventilation (Vt 7 mL∙kg^−1^, PEEP 5 cmH_2_O, Respiratory Rate (RR) 35 min^−1^) initiated as was done previously [12]. Right carotid artery (series I and II) and right femoral artery (series III) were cannulated for arterial pressure monitoring and blood gas sampling. A 7 Fr Thermodilution Balloon catheter was inserted into the Pulmonary Artery (PA) via the Right External Jugular vein. An oesophageal balloon catheter (Nutrivent; Sidam, Mirandola, Italy) was inserted [12]. A catheter (SPR-407 Mikro-Tip; Millar, Houston, TX) was inserted into the left ventricle via the left carotid artery. Airway (Paw), oesophageal (Pes), arterial, and pulmonary artery pressures were recorded at end-expiration and end-inspiration during an expiratory and inspiratory hold manoeuvre, respectively, while the left ventricular end-diastolic pressure was recorded during end-expiratory hold only (LabChart, Version 3.1, ADInstruments, Colorado Springs, USA). Cardiac Output (CO) was measured by Thermodilution Computer (Model-9520-A, Edwards Life Sciences Inc, Mississauga, Canada) in the first two series and by Transpulmonary thermodilution (PiCCO, Getinge, Sweden) in the third series.

### Abrupt PEEP removal: high versus low (series I)

This experiment was designed to assess whether lung injury could result from one single large abrupt deflation in a large mechanically ventilated animal model. The aim was to reproduce the effects of abrupt deflation previously seen in the rat model. Pilot experiments showed that increasing PEEP to 26 cmH_2_O resulted in significant cardiovascular depression (low systemic blood pressure; Additional file 1: Fig. S2). Additionally, to maximize the effects of deflation, a large difference in PEEP levels between the two groups was chosen. Six animals received a gradual increase in PEEP up to 26 cmH_2_O (3 cmH_2_O/10 min), followed by abrupt removal of PEEP and ventilation at zero end-expiratory pressure (ZEEP) for 30 min. An additional four animals (Low PEEP group) received ventilation at 5 cmH_2_O PEEP for the period of inflation, followed by abrupt change to ZEEP and ventilation at ZEEP for 30 min (Additional file 1: Fig. S1). Respiratory, hemodynamic and ABG parameters were measured throughout the experiment. Lung wet to dry ratio was measured using the right middle lobe and bronchoalveolar lavage (BAL) collected (60 mL × 3 times) from the right lower lobe (via bronchoscopy) and used for measurement of protein and IgM.

### Abrupt versus Gradual PEEP removal (series II)

The aim of this series was to study the effect of abrupt versus gradual deflation on the pulmonary vascular resistance. PEEP 26 cmH_2_O in Series I had induced significant increase in driving pressure (i.e. plateau pressure—PEEP; Additional file 1: Fig. S1) and hemodynamic depression (Additional file 1: Fig. S2), therefore, we chose to use a slightly lower PEEP for inflation limb in this series. Five pigs were crossed over randomly to either an abrupt or a gradual removal of PEEP from 20 cmH_2_O to ZEEP after a gradual increase in PEEP from zero to 20 cmH_2_O (Additional file 1: Fig. S3). After the first (gradual or abrupt) PEEP removal the pig underwent a recruitment manoeuvre, and the second removal procedure was done. Respiratory, hemodynamic and ABG parameters were measured through the experiment. The points marked with blue arrows in Additional file 1: Fig. S3 were compared.

### Fluid overload and Endo-tracheal Tube (ETT) disconnect (series III)

While the previous two series were designed as proof-of-concept studies, in this series we wanted to study the possibility of lung injury or oedema due to repeated deflations (i.e., repeated endo-tracheal disconnections) from clinical levels of PEEP in a pre-clinical model of fluid overload under mechanical ventilation. We first studied 3 ‘pilot animals’ without fluid overload (Additional file 1: Fig. S4), after which we decided to add relative fluid overload as an additional clinically relevant risk factor in order to further amplify lung vascular hydrostatic pressure as a driver of lung oedema and injury in this scenario (as shown in our previous paper; [4]). Twelve pigs were first ventilated on baseline parameters (Vt 7 mL∙kg^−1^, RR 35 breaths∙min^−1^, FiO_2_ 0.21, PEEP 10 cmH_2_O; Additional file 1: Table S1 and Fig. S5) after which they received a fluid bolus of 30 mL∙kg^−1^ and continued fluid infusion at 30 mL∙kg^−1^∙h^−1^ throughout the entire experiment. In parallel, PEEP was increased to 15 cmH_2_O and ventilation continued for 3 h. They were randomized into two groups: Disconnect (D/C, *n* = 6, ETT disconnected every 15 min for 15 s) and Control (*n* = 6, no disconnections). Respiratory, hemodynamic and ABG parameters were measured pre-disconnect in the DC group and at similar time points in the Control group. End-expiratory lung volume was directly measured on the ventilator (Carespace, GE Healthcare, Chicago, USA). Lung wet-to-dry weight ratio and BAL were analysed as in previous series, and the right lungs were excised and prepped for histology.

### Biological parameters

Histology was performed in 10 animals from series III (*n* = 5/group). Samples from the ventral regions of the excised lungs right lobe were formalin fixed, embedded in paraffin, and Haematoxylin/Eosin stained before being digitized with a brightfield scanner (Pannoramic 250 Flash II, 3DHISTECH, Budapest, Hungary). 8 captions (magnification 20x) per animal were selected randomly from different regions of the entire section. The slides were scored by a blinded accessor, for total number of alveoli, number of alveoli with alveolar oedema, alveoli with interstitial oedema, alveoli with haemorrhage and alveoli with polymorphonuclear infiltration [13]. Percent of each category (eg. % Alveolar oedema = alveoli with oedema/total alveoli × 100) was calculated, and a score was developed, where in 0% = 0; 1–25% = 1; 26–50% = 2; 51–75% = 3; 76–100% = 4. A composite score of lung injury was taken as the average of all scores for each caption and 8 captions were averaged for each animal. Automated whole slide morphometry was conducted based on a previously described protocol [14, 15]. Digitized slides were automatically partitioned into 498 × 498 μm^2^ tiles using Pannoramic Viewer (3DHISTECH, Budapest, Hungary) yielding approximately 220–1300 tiles per slide composed of 2048 × 2048 pixels each. Tiles were imported into MATLAB (MathWorks, Natick, MA) and individually converted into CIE-Lab colour space as previously described [14, 15]. Pixels were then classified as air, tissue, oedema, or red blood cells (RBCs) using a combination of two-dimensional colour-based *k*-means clustering and manual clustering seed adjustment (Additional file 1: Fig. S7). The percentage area of tissue, RBCs, oedema, and airspaces (PTA, PRA, PEA, PAA, respectively) was calculated by enumerating pixels of each type and dividing by the total number of pixels on each slide. Tile parameter values were averaged to obtain a value for each whole slide, and slides were subsequently averaged to obtain a single value for each specimen. Morphometric parameter distributions were then compared across groups to identify differences in structural properties.

Cytokines were measured in the BAL using a premixed porcine cytokine magnetic bead multiplex panel (Millipore Sigma, St Charles, USA). All samples were run in duplicate with overnight incubation. IgM in BAL was measured using an IgM Pig ELISA kit (Abcam, Cambridge, UK) and all samples were assayed in duplicates with standard curve for each analyte. The cytokine and IgM studies were done at the Analytical Facility of Bioactive Molecules (AFBM), Hospital for Sick Children, Toronto.

### Statistics

Data were analysed using Systat software Inc., Sigmaplot 12.0, UK. They were expressed as mean ± SD and compared using 1-way or 2-way ANOVA with repeated measures, followed by Student Newman-Keuls or Sidak-Holm post hoc tests for multiple comparisons. Repeated measures ANOVA on ranks was used, if either the normality or equal variance test failed. Statistical significance was set at *P* < 0.05.

## Results

### Series I: Deflation from High versus Low PEEP

Six pigs were subjected to incremental PEEP from 5 to 26 cmH_2_O, followed by abrupt release to ZEEP (High PEEP group); and 4 pigs underwent ventilation at PEEP 5 cmH_2_O followed by deflation to ZEEP (Low PEEP group). Increase in PEEP to 26 cmH_2_O caused an increase in plateau but also in driving pressure. Deflation to ZEEP reduced the plateau pressure which then gradually increased over the next 30 min of ZEEP ventilation (Additional file 1: Fig. S1A). The plateau pressure in the low PEEP group reached similar levels as in the high PEEP group after 30 min of ZEEP ventilation (Additional file 1: Fig. S1B).

**Fig. 1:**
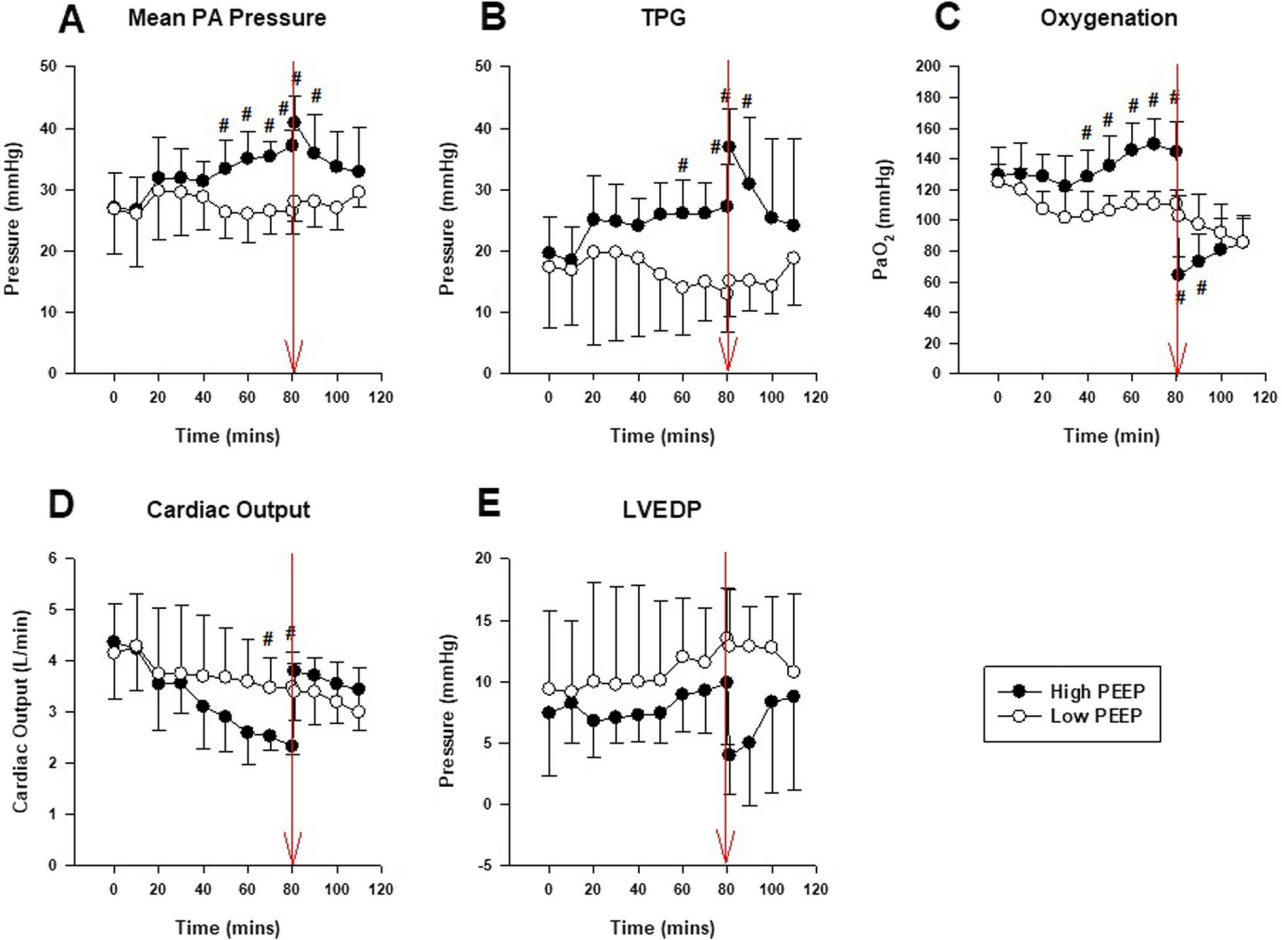
Hemodynamics and oxygenation during high versus low PEEP deflation (series I). Normal Pigs (*n* = 6) either received an increase in PEEP from 5 to 26 cmH_2_O followed by instantaneous deflation to 0 cmH_2_O (High PEEP group; closed circles) or continuous ventilation at PEEP 5cmH_2_O and deflation to 0 cmH_2_O at the same time point (Low PEEP group; open circles). The point of deflation is indicated by the red arrow. In the High PEEP group, increase in PEEP led to a corresponding increase in mean PA (**A**) with no change in LVEDP (**E**); at time of deflation the mean PA rose transiently and LVEDP decreased, thereby increasing the TPG (**B**). This was not observed in the low PEEP group. The cardiac output (**D**) in high PEEP group decreased with increasing PEEP but returned to the control levels after release of PEEP. PaO_2_ increased (**C**) with increasing PEEP and significantly decreased after PEEP removal. At 30 min after deflation PaO_2_ was similar in both groups. #*P* < 0.05, post-hoc *t test* High PEEP versus Low PEEP. Abbreviations: *PEEP* positive end-expiratory pressure, *PA* pressure pulmonary artery pressure, *TPG* transpulmonary gradient, *LVEDP* left ventricular end-diastolic pressure

In the high PEEP group, increase in PEEP led to a corresponding increase in mean pulmonary artery pressure (PAP) (Fig. 1A). Rapid deflation from high (26 cmH_2_O) PEEP caused a small transient increase in mean PAP (Fig. 1A), an abrupt increase in cardiac output (Fig. 1D) and a non-significant decrease in LVEDP (Fig. 1E) compared to deflation from low (5 cmH_2_O) PEEP (open circles; Fig. 1A, D, E). The transpulmonary gradient (TPG; mean PAP—LVEDP) was higher at deflation from high compared to low PEEP (Fig. 1B). Cardiac output at deflation was similar in both groups (Fig. 1D). Abrupt deflation from high PEEP (26 to 0 cmH_2_O) led to transient hypoxemia which recovered over the next 10 to 20 min (Fig. 1C).

Deflation after sustained inflation (to PEEP 26 cmH_2_O) led to an increase in lung water: wet-to-dry ratio in the high PEEP versus low PEEP groups was 7.1 ± 0.31 versus 6.4 ± 0.04 (*P* = 0.004). There was no difference in BAL protein content (High PEEP median 0.8 [0.7–1.3] mg/dL versus low PEEP 0.9 [0.4–1.2] mg/dL; *P* = 0.914), and IgM content (High PEEP median 5200 [3600–25000] ng/mL versus low PEEP median 9400 [3000–16000] ng/mL; *P* = 0.762).

### Series II: Abrupt versus Gradual deflation

**Fig. 2:**
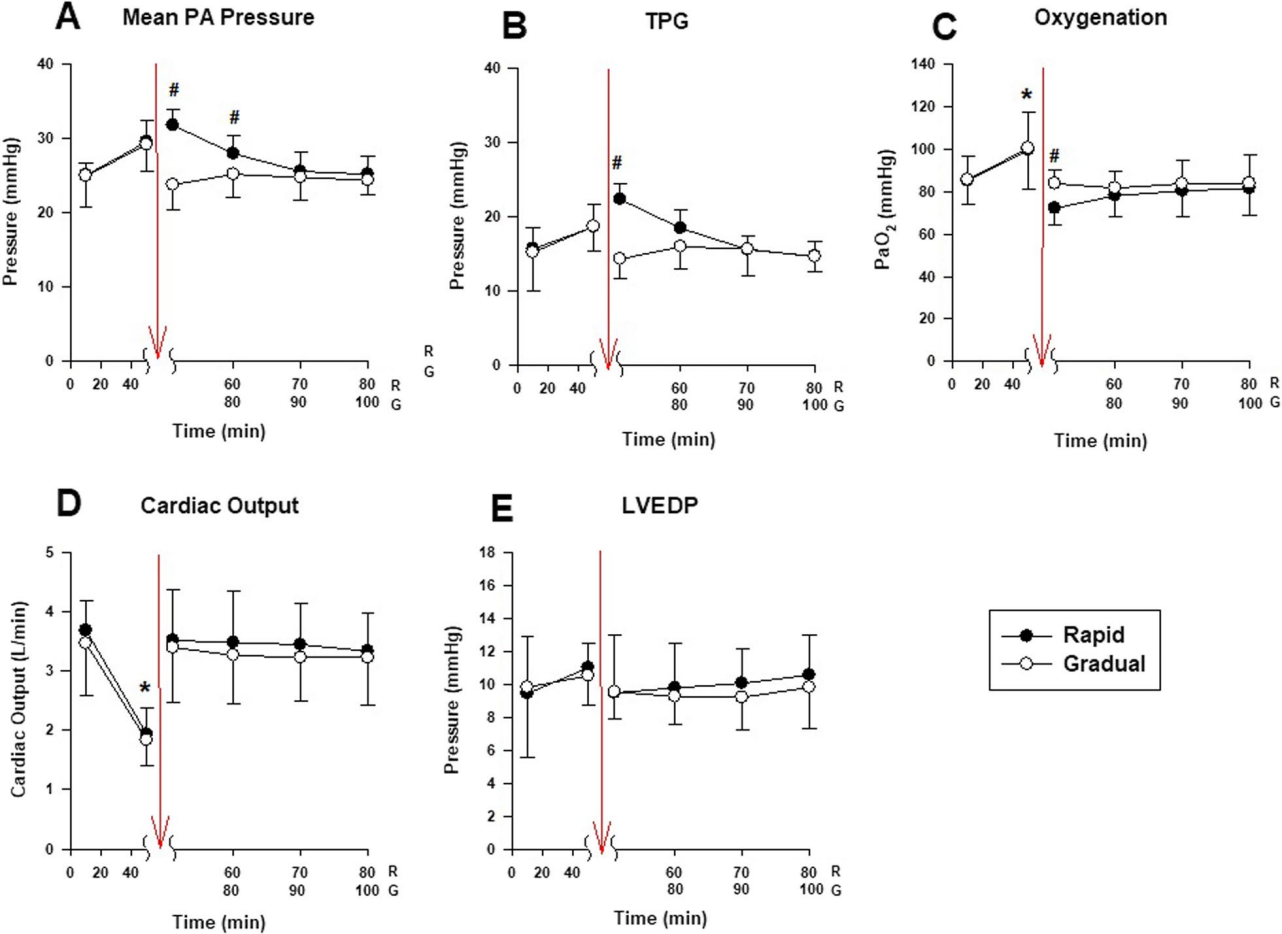
Hemodynamics and oxygenation during rapid versus gradual PEEP removal (series II): In normal pigs (*n* = 5), after stepwise increase in PEEP from 0 to 20 cmH_2_O, in a random cross-over fashion, PEEP was either dropped instantaneously to 0 (Rapid group; closed circles) or gradually at 1 cmH_2_O/min (Gradual group; open circles). Right after deflation (51 min for Rapid and 71 min for Gradual), the mean PA pressure (**A**) and TPG (**B**) were higher, and oxygenation lower (**C**) in the Rapid group, whereas the cardiac output (**D**) and LVEDP (**E**) were similar. At PEEP 20 cmH_2_O (50 min for both groups), the cardiac output was lower (**D**) and oxygenation higher (**C**) in both groups compared to other time points. #*P* < 0.05, post-hoc *t*-test Rapid versus Gradual; *P < 0.05, post hoc *t*-test 50 min versus rest of time points. Abbreviations: *PEEP* positive end-expiratory pressure, *PA* pressure pulmonary artery pressure, *LVEDP* left ventricular end-diastolic pressure, *PaO*_*2*_ partial pressure of arterial oxygen, *ZEEP* zero end-expiratory pressure, *R* Rapid Deflation group, *G* Gradual Deflation group

Five pigs received abrupt or gradual deflation in random crossover design. Upon deflation from PEEP 20 to 0 cmH_2_O, the mean PAP immediately after PEEP release was significantly higher in the rapid deflation group (Fig. 2A), with no difference in LVEDP between both groups (Fig. 2E), resulting in a higher TPG higher in the rapid deflation group (Fig. 2B). Cardiac output was similar in both groups (Fig. 2). Abrupt deflation from PEEP 20 to 0 cmH_2_O led to transient hypoxemia which recovered over the next 10 to 20 min whereas gradual deflation prevented this oxygenation deficit (Fig. 2C).

### Series III: repeated disconnections

#### Pilot animals without fluid overload

In three animals without fluid overload, repeated ETT disconnections from a PEEP of 15 cmH_2_O led to an increase in driving pressure (Additional file 1: Fig. S4A) accompanied by a small reduction in end-expiratory lung volume (Additional file 1: Fig. S4B). The maximum effect was noted within the first disconnects. PAP increased with initial disconnects and stayed elevated with constant LVEDP (Additional file 1: Fig. S4C) and decreasing cardiac output (Additional file 1: Fig. S4D). The PaO_2_ (Additional file 1: Fig. S4E) showed an initial yet transient decrease. The lung wet-to-dry ratio was 6.7 ± 0.3.

#### Randomized trial in a model of fluid overload

**Fig. 3:**
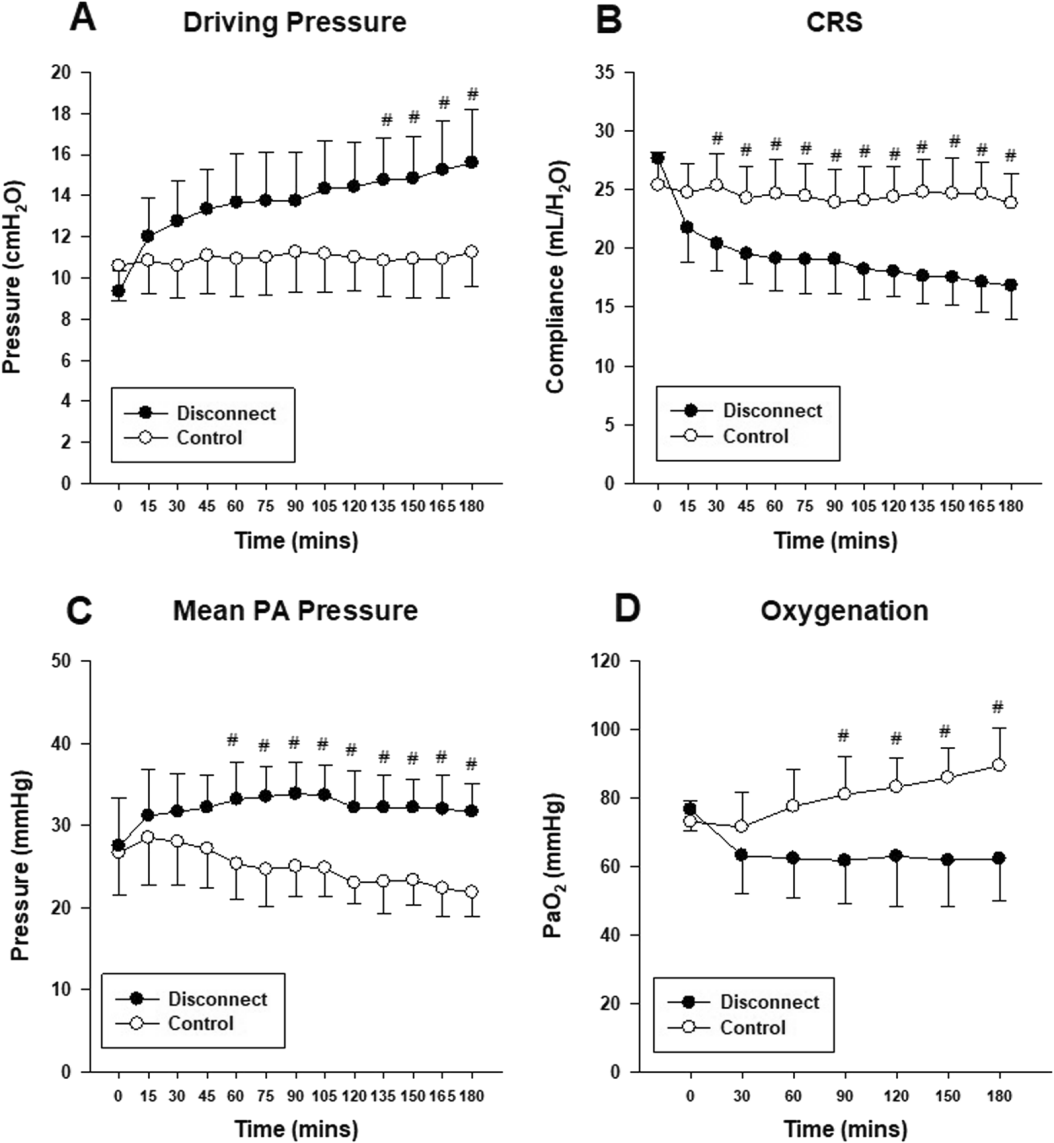
Respiratory, hemodynamic and gas exchange variables during ETT disconnect (series III): Normal Pigs (*n* = 6), after receiving volume overload were randomized to either repeated ETT disconnection every 15 min, lasting 15 s each (Disconnect group) or no disconnections (Control group), for 3 h. Repeated disconnection led to higher driving (**A**) and mean PA pressures (**C**), as well as lower respiratory system compliance (**B**) and oxygenation (**D**). # *P* < 0.05; post-hoc *t*-test; disconnect versus control. Abbreviations: *ETT* endo-tracheal tube, *CRS* compliance of the respiratory system, *PaO*_*2*_ partial pressure of arterial oxygen, *PA* pressure pulmonary artery pressure

After delivering a fluid bolus and starting fluid maintenance, the 12 animals were ventilated with PEEP 15 cmH_2_O, and were randomized to either receive repeated ETT disconnections (DC; *n* = 6) or none (Control; *n* = 6) (Additional file 1: Fig. S5). At the start of the experiment (0 min), pulmonary and hemodynamic parameters of both groups were similar (Table 1). Repeated deflations from ETT disconnects led to higher driving pressure (Fig. 3A; Table 1) and lower respiratory system compliance (Fig. 3B; Table 1). This was accompanied by progressively lower end-expiratory transpulmonary pressure (P_AW_ − P_ES_; Additional file 1: Fig. S6A) in the disconnect group despite a similar change in end-expiratory lung volume (Additional file 1: Fig. S6B).

**Table 1:** Pulmonary, hemodynamic and gas exchange variables at start and end of experiment (series III)

	Disconnect (*n* = 6)		Control (*n* = 6)	
	0 min	180 min	0 min	180 min
Plateau pressure (cmH_2_O)	24.3 ± 1.0	30.6 ± 2.6*^Ɨ^	25.6 ± 1.7	26.2 ± 1.7
Driving pressure (cmH_2_O)	9.3 ± 1.0	15.6 ± 2.6*^Ɨ^	10.6 ± 1.7	11.2 ± 1.7
C_RS_ (mL/cmH_2_O)	27.6 ± 1.8	17 ± 2.8*^Ɨ^	25.4 ± 2.8	24.0 ± 2.5
EELV (mL)	3135 ± 354	2021 ± 457^Ɨ^	3440 ± 625	2395 ± 226^Ɨ^
Mean FAP (mmHg)	83 ± 6	95 ± 10	90 ± 15.7	100 ± 20
CVP (mmHg)	11.0 ± 4.0	16 ± 5.0	11.0 ± 3.0	14.0 ± 5
Mean PAP (mmHg)	27.5 ± 5.7	32 ± 3.8*^Ɨ^	26.6 ± 5.2	21.8 ± 2.9^Ɨ^
PAWP (mmHg)	14.8 ± 1.2	17.4 ± 4.5	13.0 ± 3.1	14.6 ± 2.8
LVEDP (mmHg)	10.7 ± 3.3	17.5 ± 5.0^Ɨ^	10.5 ± 1.8	14.3 ± 3.7^Ɨ^
Cardiac output (L/min)	5.2 ± 0.2	5.3 ± 0.4	5.3 ± 0.6	4.9 ± 0.3
PVR (dynes/sec/cm^−5^)	255.5 ± 78.2	213.3 ± 72.2	247.0 ± 92.7	122.0 ± 88.6^Ɨ^
pH	7.24 ± 0.04	7.2 ± 0.05	7.24 ± 0.03	7.27 ± 0.04
PaO_2_ (mmHg)	76.5 ± 6.0	62 ± 12*^Ɨ^	73 ± 6.1	89 ± 11^Ɨ^
PaCO_2_ (mmHg)	59.4 ± 3.7	63 ± 10	62.4 ± 6.0	58 ± 7.0

Pigs with volume overload were ventilated on PEEP 15 cmH_2_O and then randomized to either receive 15 s ETT disconnects every 15 min (Disconnect group) or no disconnects (Control group); for 3 h. Pulmonary, hemodynamic and gas exchange parameters were compared at start (0 min) and end (180 min) between both groups

*ETT* endo-tracheal tube, *C*_*RS*_ Compliance of the respiratory system, *EELV* end-expiratory lung volume, *FAP* femoral arterial pressure, *CVP* central venous pressure, *PAP* pulmonary arterial pressure, *PAWP* pulmonary artery wedge pressure, *LVEDP* left ventricular end-diastolic pressure

*P < 0.05 *t*-test Disconnect versus Control; ^Ɨ^*P* < 0.05 *t*-test 0 min versus 180 min

**Fig. 4:**
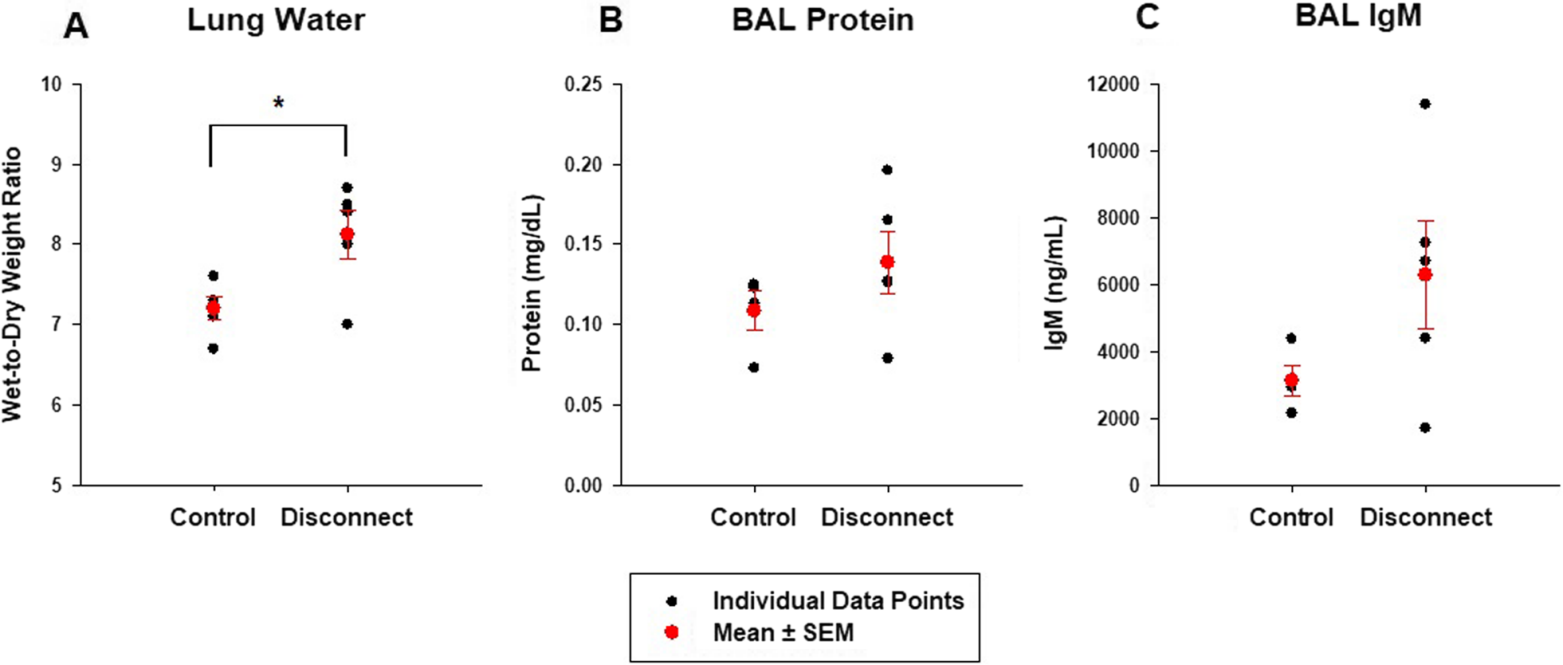
Biological outcomes during ETT disconnect (series III). Normal pigs (*n* = 6), after volume overload, were randomised to either repeated endo-tracheal tube (ETT) disconnection every 15 min, lasting 15 s each (Disconnect group) or no disconnections (Control group), for 3 h. The wet-to-dry weight ratio (lung water; **A**) was higher in the Disconnect versus Control. BAL protein (**B**) and IgM (**C**) content appeared higher in disconnect versus control but were not statistically significant. *P < 0.05; *t*-test; disconnect versus control

Repeated ETT disconnections with volume overload led to an early increase in PAP, whereas PAP progressively decreased in the control group (Fig. 3C; Table 1). There was no change in cardiac output in both groups over the course of the experiment (Additional file 1: Fig. S6C; Table 1), and LVEDP increased in both groups equally (Additional file 1: Fig. S6D; Table 1). The PVR remained unchanged in the DC group but significantly decreased in the control group (Table 1). At 180 min, PVR was higher in the DC group compared to Control, yet without reaching statistical significance (Disconnect versus Control at 180 min, *t*-test, *P* = 0.079) (Table 1). Repeated ETT disconnections led to worsening hypoxemia, whereas oxygenation improved in the absence of disconnections (Fig. 3D; Table 1). The lung wet-to-dry ratio was higher in the repeated disconnect group (Fig. 4A). Protein (Fig. 4B), IgM ((Fig. 4C) and cytokine (Additional file 1: Table S2) content in BAL were not statistically different between both groups.

**Fig. 5:**
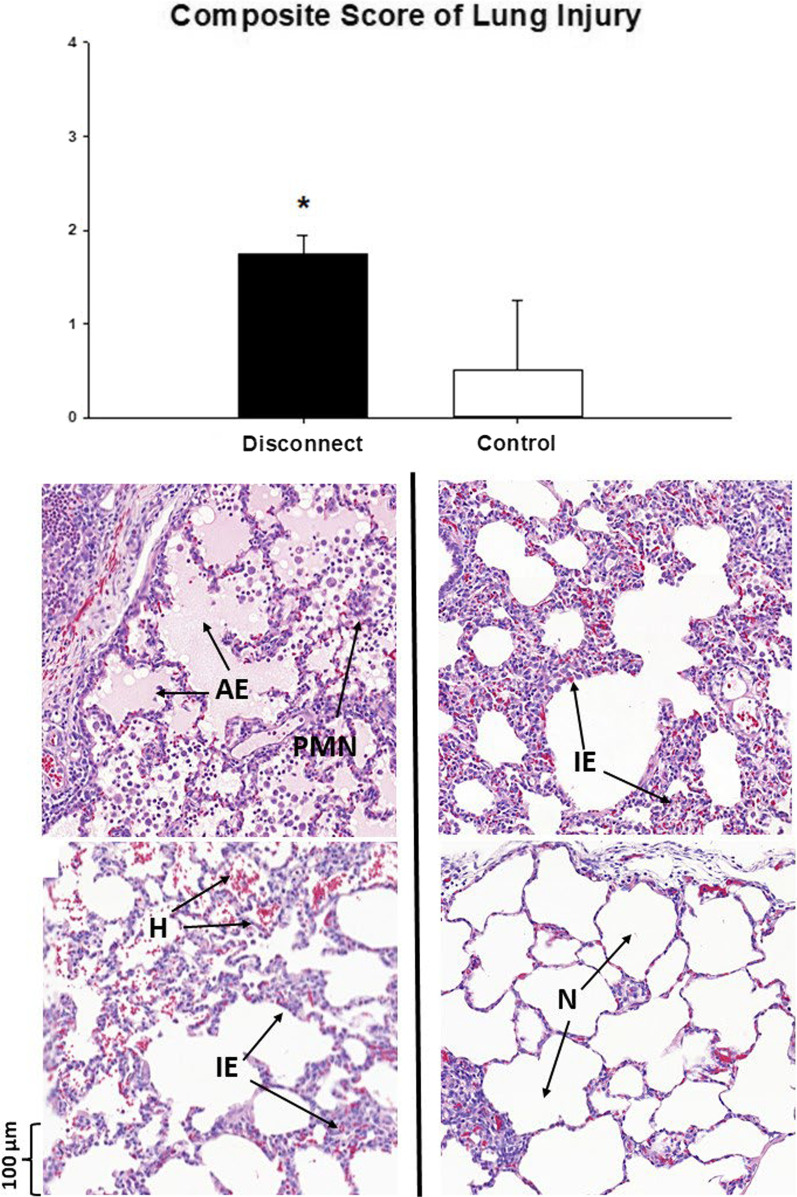
Lung Injury on Histology (series III). Normal pigs received volume overload and were randomized to either repeated endo-tracheal tube (ETT) disconnection every 15 min, lasting 15 s each (Disconnect group) or no disconnections (Control group), for 3 h. The resulting changes in Histology of ventral lung regions were scored for presence of oedema, haemorrhage, and inflammation. The median composite score of such injury was higher in Disconnect animals compared to Control (40 slides/group, *n* = 5/group). The histology image (× 20) with examples of Alveolar Oedema (AE), Interstitial Oedema (IE), Haemorrhage (H), Polymorphonuclear (PMN) infiltration and normal (N) alveoli are shown

Manual histological analysis revealed higher alveolar oedema, interstitial oedema, haemorrhage and PMN infiltration in the animals who were disconnected (Additional file 1: Table S3). The median composite score of lung injury was higher in the disconnect group versus control (Fig. 5). Similarly, the automated morphometric analysis of the ventral lung demonstrated increased PTA + PEA, increased septal thickness and lower air fraction in the disconnect group compared to the control (Table 2).

**Table 2:** Morphometric analysis of histology (series III)

	Disconnect	Control	*t*-test	Rank-Sum test*
Fractal dimension	1.73 ± 0.05	1.69 ± 0.06		0.202
MLI (μm)	111.0 ± 31.0	82.0 ± 16.0	0.057	
MLI* (μm)	74.0 ± 18.5	60.0 ± 9.4	0.111	
Septal thickness (μm)	37.0 ± 15.0	22.0 ± 7.0		**0.030**
PTA (%)	37.0 ± 12.0	23.0 ± 5.0	**0.038**	
PEA (%)	14.0 ± 4.0	11.0 ± 4.0	0.358	
PTA + PEA (%)	51.0 ± 15.0	33.0 ± 9.0	**0.026**	
PRA (%)	3.5 ± 1.4	2.9 ± 1.5	0.501	
PAA (%)	45.0 ± 14.0	64.0 ± 10.0	**0.025**	
Airspace density (airspaces/mm^2^)	3.6 ± 0.2 × 10^−5^	4.0 ± 0.2 × 10^−5^	0.320	

After 3 h of fluid overload and either repeated ETT disconnects (Disconnect Group) or none (Control), the morphometric analysis in the ventral lung regions revealed higher oedema and lower air fraction with repeated disconnects. *t*-test Disconnect versus Control. *Rank sum test was done when normality failed. Bold value: P < 0.05 was considered significant

*MLI* mean linear intercept, MLI* MLI − Septal thickness, *PTA* percentage tissue area, *PEA* percentage oedema area, *PRA* percentage RBC area, *PAA* percentage air area

## Discussion

Several novel and physiologically relevant results were observed in this study. Abrupt deflation from high PEEP generates pulmonary oedema, increased pulmonary vascular pressure and hypoxemia, while gradual deflation prevents this from occurring. Repeated disconnections from a clinically relevant level of PEEP lead to an increase in lung water (pulmonary oedema), worsening respiratory function (reduced compliance and hypoxemia), and increased right ventricular afterload (high pulmonary artery pressure). Most of these changes are observed within the first few disconnections but are sustained for > 3 h, whereas animals exposed to no disconnections do not exhibit any of these phenomena.

### Effect of a single deflation

*Series I* was designed with the intention of validating our previous observations in the rat model [4], wherein abruptly deflating from PEEP of 12 cmH_2_O led to pulmonary oedema, cellular and protein leak. This injury was thought to result from abruptly increased forward flow (vascular surge) coupled with increased backward pressure (raised LVEDP). In the current model, rapid release from high PEEP resulted abruptly in lower oxygenation, which steadily recovered over the next 30 min and higher lung wet-to-dry ratio, while protein and IgM leak in the BAL remained unchanged. Together this suggests increased lung water and hydrostatic pulmonary oedema. A plausible mechanism for the formation of this oedema can be inferred from the hemodynamic events following deflation. The abrupt release in airway pressure from high PEEP increases biventricular preload and cardiac output [4], leading to an abrupt surge in vascular flow. Additionally, the abrupt release in airway pressure from high PEEP further increased pulmonary artery pressure. This increase in pulmonary artery pressure was not due to an increase in left ventricular end-diastolic pressure contrasting with the findings in the rodent model [4]. Although the rise in pulmonary artery pressure could be due to the increase in cardiac output, cardiac output following rapid deflation did not exceed baseline values (i.e. @PEEP 5cmH_2_O) or those of the low PEEP control at deflation, during which the pulmonary artery pressure was lower. This indicates that the rise in pulmonary artery pressure (and transpulmonary gradient) is independent of increased forward flow or backward pressure suggesting increased pulmonary vascular resistance. Although, the current data cannot discriminate between the anatomical locations of increased PVR (arterial vs. microvascular vs. venous), the occurrence of pulmonary oedema suggests either microvascular or pulmonary venous origins of increased PVR. The microvascular site can be potentially explained by decruitment of interalveolar corner vessels during rapid deflation. Possibly, rapid deflation from high PEEP could also cause transient atelectasis with resultant hypoxic pulmonary vasoconstriction and increased PVR. The pulmonary vascular surge coupled with the higher PVR results in water leak into the lungs and hydrostatic pulmonary oedema as shown by increased lung wet-to-dry ratio in the high PEEP group. Independent of the exact mechanism, our findings are in keeping with previous data demonstrating that high pulmonary vascular flow or surge can induce oedema. High pulmonary vascular flow and pulmonary artery pressure have been shown to cause haemorrhagic oedema in *ex-vivo* models [16, 17]. Moreover, pulmonary vascular surge after cardiovascular surgeries has led to altered respiratory function and oedema [18]. Sudden removal of positive pressure ventilation can increase left ventricular afterload especially in the failing heart and if coupled with volume overload can result in weaning induced pulmonary oedema [19–21]. Treatment with diuretics in such conditions enables successful weaning [22].

In *Series II*, a gradual deflation prevented the increase in pulmonary artery pressure, suggesting that rapidity of airway pressure release triggers the increase in PVR. Reduced vascular surge in turn probably contributes to the fact that gradual deflation prevented the transient oxygenation deficit. Additionally, despite being on ZEEP, oxygenation did not lower with gradual deflation; this could be the result of a well recruited lung before deflation.

### Repeated ETT Disconnection and Volume overload

Repeated endo-tracheal tube disconnection (and therefore repeated lung deflations) in the presence of volume overload, led to deterioration of respiratory mechanics (increase in driving pressure and lowered respiratory system compliance). The biggest changes in respiratory mechanics were seen after the first few disconnects. This was similar to the observation of an early drop in oxygenation which persisted. At the end, the animals undergoing repeated disconnections demonstrated higher lung wet-to-dry ratio, increased proportion of lung tissue section area occupied by tissue and oedema (i.e. PTA + PEA) on morphometry and the presence of alveolar and interstitial oedema on histology. Together these results suggest worsening respiratory function (mechanics and oxygenation) from ongoing pulmonary oedema in the disconnect group. In the control group (volume overload without disconnections) oxygenation improved and compliance stayed stable despite worsening end-expiratory lung volume. The two groups received the same amount of fluid infusions and the lung wet-to-dry weight ratio, oedema fraction and injury score were higher in the deflation as compared to the control group, showing the specific impact of disconnection. Increase in lung water has also been shown to induce inflammation [23].

Volume overload is a very frequent situation in ICU patients. In septic shock, it has been shown that 86 percent of patients have a positive fluid balance, and 35% had volume overload upon ICU discharge [24]. It is associated with worse functional outcomes [25]. Based on the hemodynamic origin of deflation-induced lung oedema [4], we considered this frequent situation could expose to a higher risk of complications induced by disconnection. While increase in driving pressure and PAP occurred in the pilot experiments without volume overload, these were likely from atelectasis alone, as can be concluded from the lowered end-expiratory lung volume without increased lung wet-to-dry weight ratio.

Attesting to the clinical relevance of our findings, changes in respiratory function, associated with endo-tracheal tube disconnection, have been previously observed in clinical studies. Maggiore and colleagues reported that suctioning associated with tube disconnection led to alveolar decruitment, lower lung compliance and caused hypoxemia. These changes could be reduced by performing recruitment manoeuvres during suctioning episodes [6]. Endo-tracheal tube suctioning episodes (which involved tube disconnection) are frequently associated with hypoxemia, heart rate and blood pressure changes, as well as haemorrhagic secretions [7]. In a systematic review it was noted that suctioning episodes resulted in several adverse events including hypoxia, bradycardia, increased blood pressure and atelectasis in the Pediatric and neonatal group [5]. While these findings have been largely attributed to atelectasis formation or parasympathetic activation during suctioning, our study presents an additional mechanism, namely lung oedema formation due to rapid and repeated lung deflations. Specifically during disconnects, repeated deflations may increase LV afterload by lowering pleural pressure, and concomitant surges of blood flow may then result in overall increased lung microvascular pressures and subsequent oedema formation.

Pulmonary artery pressure increased after few disconnects and stayed elevated. The persistent elevation of pulmonary artery pressure may suggest increased PVR either from ongoing oedema and/or hypoxic pulmonary vasoconstriction [26]. It has been shown that isocapnic hypoxia in healthy individuals could induce a 150% increase in PVR [27] and administration of 100% oxygen could reduce PVR by 10–15% in patients with ARDS [28]. In case of no disconnections, on the other hand, pulmonary artery pressure and PVR were reduced. Improved oxygenation can potentially explain the reduction in PVR (and therefore of PAP) in the control group. Atelectasis can also result in increased PVR and right heart failure; however, that happens over time accompanied with lung injury and without recruitment [29]. This is unlikely to be the case here as both groups had a similar loss in end-expiratory lung volume; suggesting that even the control group developed some atelectasis but with lower pulmonary vascular resistance. On the other hand, repeated disconnections in animals without volume overload resulted in increased PAP, accompanied with reduced cardiac output, suggesting increased PVR, albeit without an increase in lung water.

### Translational relevance

The finding of increased lung water and reduced oxygenation from repeated abrupt deflations has several translationally relevant implications. This increased lung water, in the model of fluid overload, does not originate from increased left ventricular end-diastolic pressure (as in heart failure) or from inflammation (as in ARDS), but is in-part caused by ventilator disconnections, becoming a potential independent contributor towards pathology and outcomes. In the absence of fluid overload, such disconnections did not result in high lung water, potentially because of progressive adaptation in cardiac output and therefore less chance of flooding the lungs. It can be safely assumed that such increase in lung water may contribute to worsening hypoxemia in patients with existing lung injury on high PEEP. Szakmany et al. [30] and Kushimoto et al. [31] found a negative correlation between P/F ratio and lung water in patients with ARDS. Total increased lung water [9, 11] and higher pulmonary vascular resistance are associated with poor outcomes in ARDS [32–34]. It is important to note that changes in respiratory mechanics, pulmonary artery pressure and oxygenation occur within the first few disconnects. Hence this study indicates the need to limit ventilator disconnections in patients on moderate to high PEEP and especially in those with pre-existing high lung water such as ARDS and heart failure. The study also highlights the importance of monitoring hemodynamics and oxygenation during such events. Changes in extravascular lung water as measured via PiCCO may be useful tools to monitor the bedside impact of abrupt decreases in ventilator pressures. Overall, this study adds to emerging evidence of vascular forces (beyond heart failure) contributing to oedema formation as indicated recently in a study of cardiopulmonary resuscitation [35].

### Limitations

First, this preclinical work of short experimental durations limits its absolute applicability. For this study we did not induce lung injury or cardiac failure, which could possibly aggravate the effect of pulmonary oedema, and would be translationally relevant. However, we believe, that worsening pulmonary oedema in ventilated lungs is indicative of a significant problem which is likely to worsen in sicker patients. Additionally, we did not use the same levels of PEEP in all three series of experiments, making it complex to directly compare observations between the series. We did not perform recruitment manoeuvres to see if outcome was reversible. The study was designed as a proof-of-concept study to evaluate the cumulative problem of repeated lung deflations. Importantly, deflation induced oedema was only evident in animals with fluid overload and not in animals without, suggesting a contribution from high circulating fluid volume. The accuracy of the automated morphometric analysis could benefit from an improved segmentation that is not dependent on manual seed adjustment. The reporting of the combined PTA + PEA is partially due to difficulty resolving interstitial oedema and components of the tissue which could potentially be resolved by a more comprehensive segmentation that accounts for additional pixel parameters besides colour.

## Conclusions

In this large animal model, single abrupt deflation from high PEEP, and repeated short deflations from moderate PEEP (during volume overload) cause pulmonary oedema, impaired oxygenation, and increased PVR, thus replicating our previous finding from rodents. Rapid deflation may thus be a clinically relevant cause of impaired lung function, which can be avoided by minimizing rapid deflations (avoiding disconnects) or by deploying gradual deflation.

## Supplementary Information

**Additional file 1.** Detailed Methods and Supplemental Results.

## Data Availability

Authors agree to make the data available.
