# Supplementary material for: Repeated endo-tracheal tube disconnection generates pulmonary edema in a model of volume overload: an experimental study
Source: Crit Care. 2022 Feb 18;26:47. doi: 10.1186/s13054-022-03924-2 (PMC8857825; doi:10.1186/s13054-022-03924-2)
Supplement: Supplementary file 1 — Additional file 1. Detailed Methods and Supplemental Results. [file 13054_2022_3924_MOESM1_ESM.docx]

**Repeated Endo-tracheal Tube Disconnection Generates Pulmonary Edema in Model of Volume Overload: An experimental study**

**Online Supplement**

**METHODS – *Long Version, Supplement***

All experiments were conducted according to the Canadian Animal Care guidelines and were approved by the Animal Care Committee at The Hospital for Sick Children, Toronto, Canada (animal use protocol # 58058).

**Animal Preparation**

27 healthy female Yorkshire pigs (36.5 ± 3.9 kg) were studied in 3 series of experiments. After sedation (Ketamine 12 mg∙kg^-1^) and general anesthesia (Pentobarbital 10 mg∙kg^-1^∙hr^-1^) surgical depth of anesthesia was confirmed (no response to hard toe pinch), and endo-tracheal intubation was performed (9 mm endo-tracheal tube). Muscle paralysis (Rocuronium bolus 1mg.kg^-1^ followed by infusion at 0.02 mg∙kg^-1^∙hr^-1^) and ventilation (Vt 7 mL∙kg^-1^, PEEP 5 cmH_2_O, Respiratory Rate (RR) 35 min^-1^) were then initiated and Vt and RR were kept constant throughout the experimental period. In the first two series, the right carotid artery was cannulated for arterial pressure measurement and arterial blood gas (ABG) sampling. In the third series, the right femoral artery was cannulated for arterial pressure measurement, ABG sampling and Pulse induced Contour Cardiac Output (PiCCO) sensing. A 7 Fr Thermodilution Balloon catheter (Arrow, Teleflex Medical, Markham, Canada) was inserted into the Pulmonary Artery (PA) via the Right External Jugular vein. An esophageal catheter (Nutrivent; Sidam, Mirandola, Italy) was inserted and the position confirmed with occlusion test. A catheter (SPR-407 Mikro-Tip; Millar, Houston, TX) was inserted into the left ventricle via the left carotid artery.

**Measurements**

Airway (Paw), esophageal (Pes), arterial, and pulmonary artery pressures were recorded at end-expiration and end-inspiration during an expiratory and inspiratory hold maneuver, respectively, while the left ventricular end-diastolic pressure was recorded during end-expiratory hold only. All pressures were measured by Powerlab (Powerlab 8/35 ADInstruments, Colorado Springs, USA) and recorded by LabChart (Version 3.1, ADInstruments, Colorado Springs, USA). Arterial blood gases were measured on the ABL800 (Radiometer, Copenhagen, Denmark). Cardiac Output (CO) was measured by Thermodilution Computer (Model -9520-A, Edwards Life Sciences (Canada) Inc, Mississauga, Canada) in the first two series and by Transpulmonary thermodilution (PiCCO, Getinge, Sweden) in the third series. Ten mL of cold saline was injected into the proximal port of the pulmonary artery catheter and readings recorded from the sensor in the arterial line. An average of three measurements were used at each point.

**Abrupt PEEP removal: high versus low (Series I)**

This experiment was designed to assess whether lung injury could result from one single large abrupt deflation in a large mechanically ventilated animal model. The aim was to reproduce the effects of abrupt deflation previously seen in the rat model. Pilot experiments showed that increasing PEEP to 26 cmH_2_O resulted in significant cardiovascular depression (low systemic blood pressure; **Figure S2**). Additionally, to maximize the effects of deflation, a large difference in PEEP levels between the two groups was chosen. Six animals received a gradual increase in PEEP up to 26 cmH_2_O (3 cmH_2_O/10min), followed by abrupt removal of PEEP and ventilation at zero end-expiratory pressure (ZEEP) for 30 min. An additional four animals (Low PEEP group) received ventilation at 5 cmH_2_O PEEP for the period of inflation, followed by abrupt change to ZEEP and ventilation at ZEEP for 30 min **(Figure S1)**. Respiratory, hemodynamic and ABG parameters were measured throughout the experiment. Lung wet to dry ratio was measured using the right middle lobe and bronchoalveolar lavage (BAL) collected (60 mL x 3 times) from the right lower lobe (via bronchoscopy) and used for measurement of protein and IgM.

**Abrupt vs Gradual PEEP removal (Series II)**

The aim of this series was to study the effect of abrupt *vs* gradual deflation on the pulmonary vascular resistance. PEEP 26 cmH_2_O in Series I had induced significant increase in driving pressure (i.e. plateau pressure – PEEP; **Figure S1**) and hemodynamic depression **(Figure S2)**, therefore, we chose to use a slightly lower PEEP for inflation limb in this series. Five pigs were crossed over randomly to either an abrupt or a gradual removal of PEEP from 20 cmH_2_O to ZEEP after a gradual increase in PEEP from zero to 20 cmH_2_O **(Figure S3)**. After the first (gradual or abrupt) PEEP removal the pig underwent a recruitment maneuver, and the second removal procedure was done. Respiratory, hemodynamic and ABG parameters were measured through the experiment. The points marked with blue arrows in **Figure S3** were compared.

**Fluid overload and Endo-tracheal Tube (ETT) disconnect (Series III)**

While the previous two series were designed as proof-of-concept studies, in this series we wanted to study the possibility of lung injury or edema due to repeated deflations (i.e., repeated endo-tracheal disconnections) from clinical levels of PEEP in a pre-clinical model of fluid overload under mechanical ventilation. We first studied 3 ‘pilot animals’ without fluid overload **(Figure S4)**, after which we decided to add relative fluid overload as an additional clinically relevant risk factor in order to further amplify lung vascular hydrostatic pressure as a driver of lung edema and injury in this scenario (as shown in our previous paper; (1)), and to justify the use of higher PEEP. They were randomized into two groups: Disconnnect and Control. Twelve pigs were first ventilated on baseline parameters (Vt 7 mL∙kg^-1^, RR 35 breaths∙min^-1^, FiO_2_ 0.21, PEEP 10 cmH_2_O; **Figure S5**). After recording the baseline parameters (shown in **Table S1**), animals received a fluid bolus of 30 mL∙kg^-1^ and continued to receive fluids at the rate of 30 mL∙kg^-1^∙hr^-1^ (normal maintenance is 7–10mL∙kg^-1^∙hr^-1^) throughout the entire experiment. In parallel, PEEP was increased to 15 cmH_2_O. Pigs were randomized to receive 3 hours of ventilation on PEEP 15 cmH_2_O with either ETT disconnected every 15 min for 15 seconds (Disconnect/DC, n=6) or no disconnects (Control, n=6). Respiratory, hemodynamic and ABG parameters were measured pre-disconnect in the DC group and at similar time points in the Control group. In addition, cardiac output was measured, and end-expiratory lung volume was directly measured on the ventilator (Carespace, GE Healthcare, Chicago, USA). These were terminal experiments; lung wet-to-dry ratio and BAL were analyzed as in previous series. The right lungs were excised and prepped for histology.

**Biological parameters**

Histology was performed in 10 animals from series III (n=5/group). Samples from the ventral regions of the excised lungs right lobe were formalin fixed, embedded in paraffin, and Hematoxylin/Eosin stained before being digitized with a brightfield scanner (Pannoramic 250 Flash II, 3DHISTECH, Budapest, Hungary) with a x20/0.8 objective at 0.243x0.243 μm^2^ per pixel. 8 captions (magnification 20x) per animal were selected randomly from different regions of the entire section. The slides were scored by a blinded accessor, for total number of alveoli, number of alveoli with alveolar edema, alveoli with interstitial edema, alveoli with hemorrhage and alveoli with polymorphonuclear infiltration (2). Percent of each category (eg. % Alveolar edema = alveoli with edema/total alveoli × 100) was calculated, and a score was developed, where in 0% = 0; 1-25% = 1; 26 – 50% = 2; 51 – 75% = 3; 76 – 100% = 4. A composite score of lung injury was taken as the average of all scores for each caption and 8 captions were averaged for each animal. Automated whole slide morphometry was conducted based on a previously described protocol (3, 4). Digitized slides were automatically partitioned into 498 × 498 μm^2^ tiles using Pannoramic Viewer (3DHISTECH, Budapest, Hungary) yielding approximately 220-1300 tiles per slide composed of 2048x2048 pixels each. Tiles were imported into MATLAB (MathWorks, Natick, MA) and individually converted into CIE-Lab color space as previously described (3, 4). Pixels were then classified as air, tissue, edema, or red blood cells (RBCs) using a combination of two-dimensional color-based *k*-means clustering and manual clustering seed adjustment. *k*-means clustering seed selection and verification of segmentation was tuned manually and features minor variations in centroid value between slides to ensure accurate segmentation **(Figure S7)**. The percentage area of tissue, RBCs, edema, and airspaces (PTA, PRA, PEA, PAA, respectively) was calculated by enumerating pixels of each type and dividing by the total number of pixels on each slide. Tile parameter values were averaged to obtain a value for each whole slide, and slides were subsequently averaged to obtain a single value for each specimen. Morphometric parameter distributions were then compared across groups to identify differences in structural properties.

Analytes shown in **Table S2** in BAL samples were measured using a pre-mixed multiplex kit purchased from Millipore Sigma (Millipore, St. Charles, MO). Analyte concentrations were measured using the porcine cytokine/chemokine magnetic bead multiplex panel (Cat. #: PCYTMG-23K-13PK) with 92% - 103% accuracy. The inter- and intra-assay precision are as followed: <20% and <10% respectively. The analytes were quantified using Luminex xMAP instruments (Luminex 200; Luminex, USA). All samples, standards and controls were assayed in duplicate with a standard curve for each analyte (see ranges below). BAL samples (50µl) for the quantification of all 13 analytes were run neat with an overnight incubation. The kit was performed according to the manufacturer’s instructions with an additional low standard point for all analytes (8-point standard curve). This multiplex kit has a quality level of MQ200 (MilliporeSigma).

IgM in BAL samples were measured using an ELISA kit purchased from Abcam (Abcam, Cambridge, UK). IgM concentrations were measured using the IgM Pig ELISA kit (Cat. #: ab190537) with an assay sensitivity of 5.072ng/mL. The inter- and intra-assay precision of the kit are both <10%. All standards and samples were assayed in duplicate with a standard curve ranging from 12.5ng/mL to 400 ng/mL. BAL samples (100µL) were diluted 1:100 using the kit sample diluent provided. The absorbance at 450nm was measured using a plate reader (SpectraMax M2, Molecular Devices) and the optical density used to quantify the IgM concentrations. The kit was performed according to the manufacturer’s instructions. The cytokine and IgM studies were done at the Analytical Facility of Bioactive Molecules (AFBM), Hospital for Sick Children, Toronto.

**Statistics**

Data were analyzed using Systat software Inc., Sigmaplot 12.0, UK. They were expressed as mean ± SD and compared using 1-way or 2-way ANOVA with repeated measures, followed by Student Newman-Keuls or Sidak-Holm *post hoc* tests for multiple comparisons. Repeated measures ANOVA on ranks was used, if either the normality or equal variance test failed. Statistical significance was set at p <0.05.

**References**

1. Katira BH, Engelberts D, Otulakowski G, Giesinger RE, Yoshida T, Post M, et al. Abrupt Deflation after Sustained Inflation Causes Lung Injury. Am J Respir Crit Care Med. 2018;198(9):1165-76.

2. Bai C, She J, Goolaerts A, Song Y, Shen C, Shen J, et al. Stress failure plays a major role in the development of high-altitude pulmonary oedema in rats. Eur Respir J. 2010;35(3):584-91.

3. Fliss JD, Zanette B, Friedlander Y, Sadanand S, Lindenmaier AA, Stirrat E, et al. Hyperpolarized (129)Xe magnetic resonance spectroscopy in a rat model of bronchopulmonary dysplasia. Am J Physiol Lung Cell Mol Physiol. 2021;321(3):L507-l17.

4. Lindenmaier A SS, Zheng J, Zanette B, Friedlander Y, Couch M, Stirrat E, Bouch S, Lok I, Ivanovska J, Post M, Jankov R.P. Santyr G. Automated whole slide morphometry (AWSM-Q): AValidation Study for Lung Histology Quantification in Rodent Models of Bronchopulmonary Dysplasia. Am J Respir Crit Care Med. 2019;199:A5492.

**FIGURES - *Supplement***

**Figure S1: Experimental Design for Rapid PEEP Removal (Series I)**


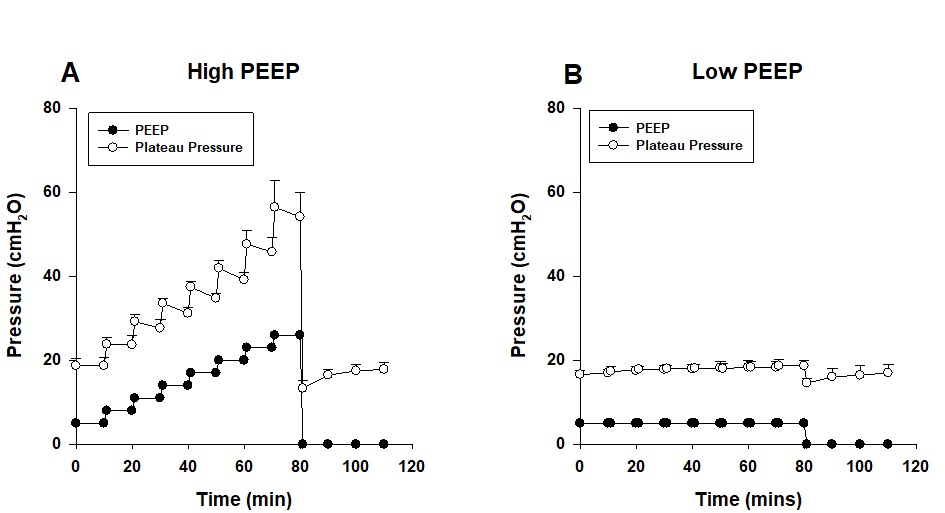


In the **High PEEP group,** normal Pigs (n=6) received an increase in PEEP from 5 to 26 cmH_2_O followed by instantaneous deflation to 0 cmH_2_O and ventilation at ZEEP for 30 min. The **Low PEEP group** received continuous ventilation at PEEP 5 cmH_2_O and deflation to 0 cmH_2_O at same time point followed by ventilation at ZEEP for 30 min. Respective changes in plateau pressures are denoted as open circles and animals were ventilated on VCV mode with V_T_ 6mL.Kg^-1^. ***Abbreviations*** PEEP Positive End-Expiratory Pressure; ZEEP Zero End-Expiratory Pressure; VCV Volume Control Ventilation; V_T_ Tidal Volume

**Figure S2: Systemic Hemodynamics Pressure during Rapid PEEP Removal (Series I)**


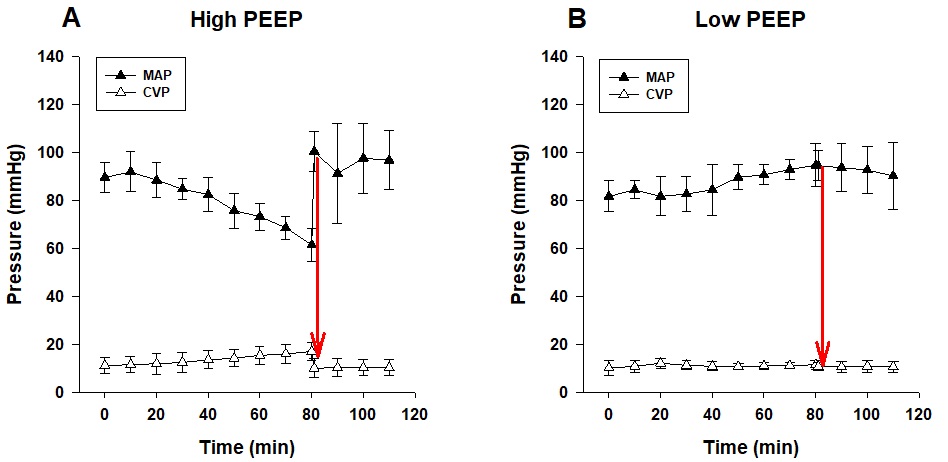


In the High PEEP group **(Panel A),** increase in PEEP led to a corresponding decrease in MAP and increase in CVP; at time of deflation the MAP rose sharply and CVP decreased. In the Low PEEP group **(Panel B),** MAP and CVP remained unchanged after deflation. ***Abbreviations*** PEEP Positive End-Expiratory Pressure; MAP Mean (Systemic) Arterial Pressure; CVP Central Venous Pressure.

**Figure S3: Experimental Protocol for Comparing Rapid and Gradual PEEP removal (Series II)**


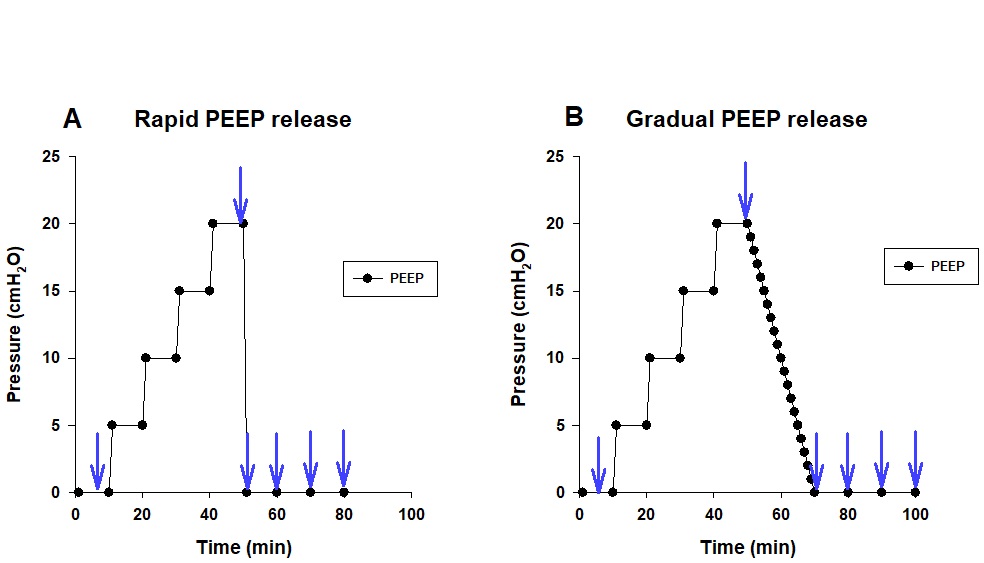


Five normal pigs, in a randomized crossover fashion, underwent ventilation in VCV mode, PEEP increments from 0 to 20 cmH_2_O followed either a rapid (instantaneous from 20 to 0 cmH_2_O) or gradual (20 to 0 cmH_2_O at 1 cmH_2_O/min) PEEP decrement. Data were compared at time points marked by arrows. ***Abbreviations*** PEEP Positive End-Expiratory Pressure; VCV Volume Control Ventilation.

**Figure S4: ETT disconnects without volume overload (Series III)**


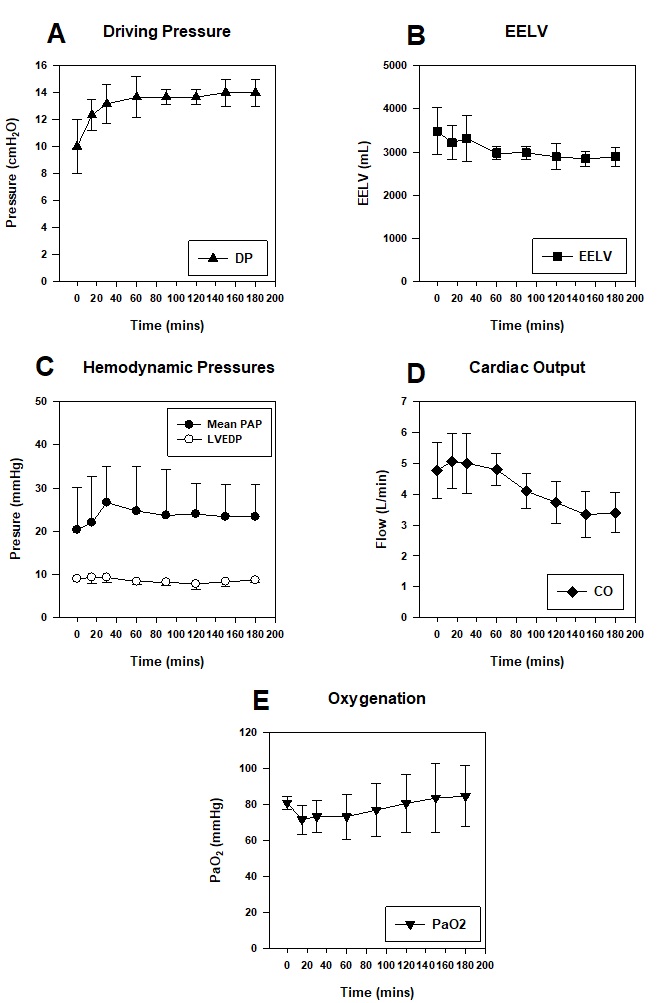


Pigs without volume overload (n=3) underwent repeated ETT disconnections from PEEP 15 cmH_2_O at every 15 min for 15 secs each. The driving pressure (**Panel A**) increased with few initial disconnects, accompanied with small decrease in EELV (**Panel B**), small increase in PAP (**Panel C**) and decrease in cardiac output (**Panel D**). The EVLW did not change (**Panel E**), and oxygenation improved after initial decrease (**Panel F**). ***Abbreviations*** DP Driving Pressure; EELV End-Expiratory Lung Volume; PAP Pulmonary Artery Pressure; LVEDP Left Ventricular End-Diastolic Pressure; CO Cardiac Output; EVLW Extra-Vascular Lung Water; PaO_2_ is Partial Pressure of Oxygen.

**Figure S5: Experimental Protocol (Series III)**


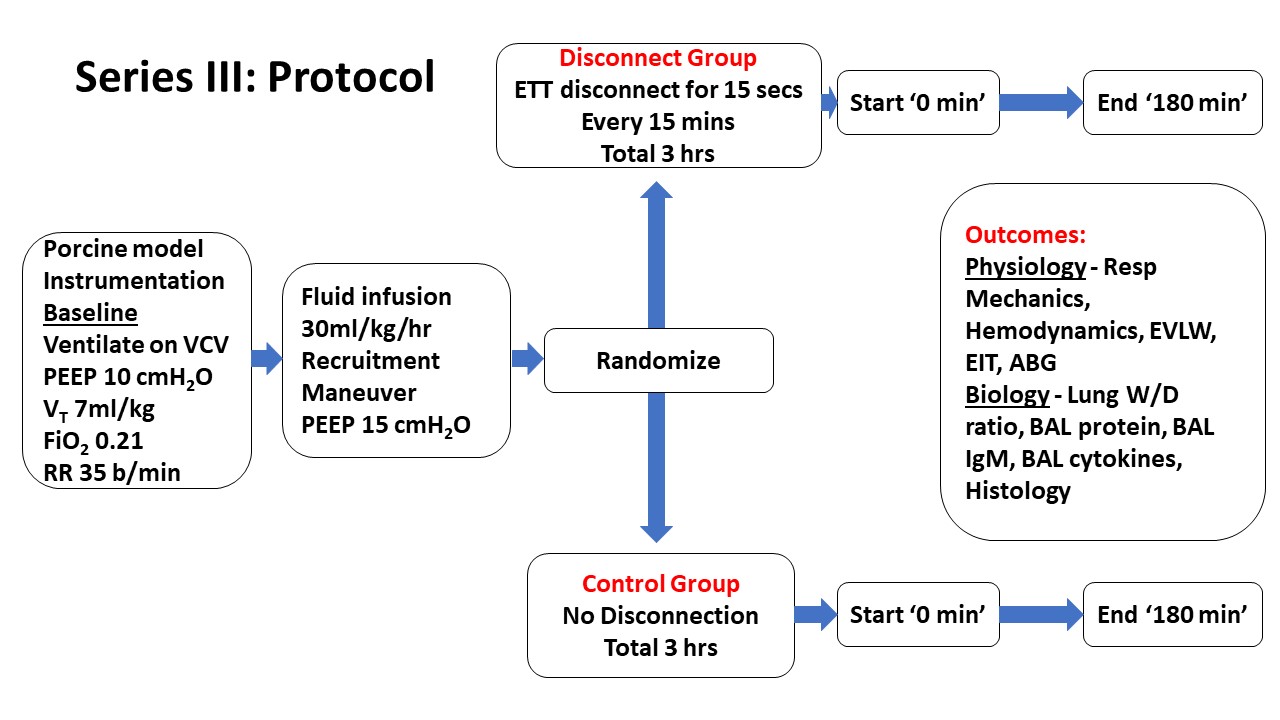


Normal Pigs (n=12), after baseline measurements (PEEP 10 cmH_2_O), received fluid infusion at 30mL/Kg/hr (volume overload) and were ventilated at PEEP 15 cmH_2_O. Thereafter they were randomized to either receive repeated ETT disconnection every 15 minutes, lasting 15 seconds each **(Disconnect group; n=6)** or no disconnections **(Control group; n=6)** for 3 hours. ***Abbreviations*** VCV Volume Control Ventilation; V_T_ Tidal Volume; RR Respiratory Rate; ETT Endo-tracheal Tube; EVLW Extra-Vascular Lung Water; EIT Electrical Impedance Tomography; ABG Arterial Blood Gases; W/D Ratio Wet-to-Dry Ratio; BAL Bronchoalveolar Lavage; IgM Immunoglobulin M.

**Figure S6: Pulmonary and Hemodynamic Variables during ETT Disconnect (Series III)**


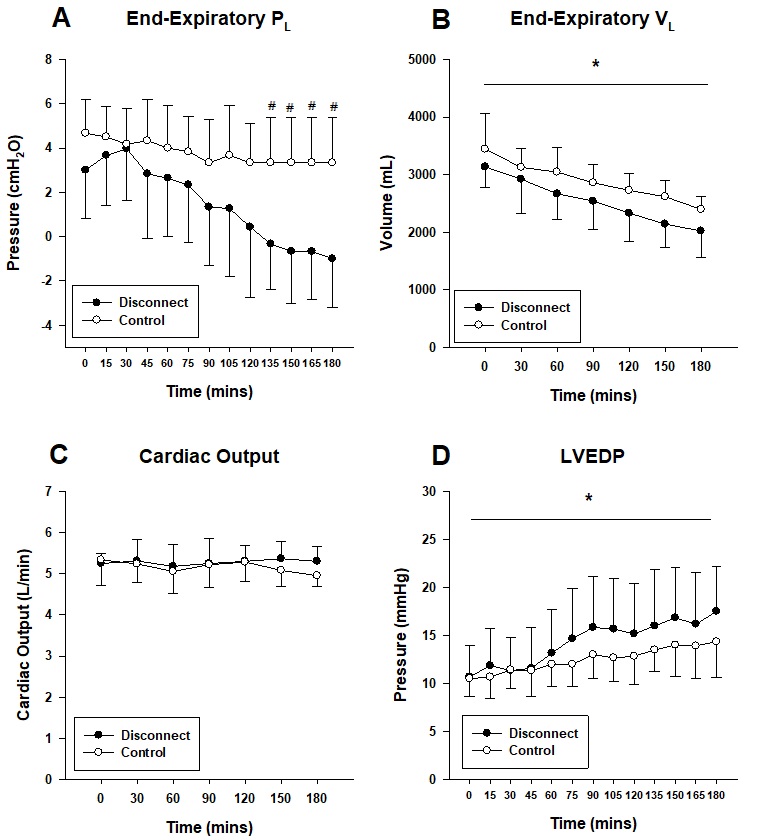


Normal Pigs (n=12), after volume overload, were randomized to either receive repeated ETT disconnection every 15 minutes, lasting 15 seconds each **(Disconnect group; n=6)** or no disconnections **(Control group; n=6)** for 3 hours. Repeated disconnections led to lower end-expiratory P_L_ **(Panel A)**, a decrease in end-expiratory V_L_ **(Panel B)**, no change in cardiac output **(Panel C)** and an increase in LVEDP **(Panel D)**. # P<0.05 Disconnect vs Control. ***Abbreviations*** ETT Endo-tracheal Tube; P_L_ Transpulmonary pressure; V_L_ Lung Volume; LVEDP Left Ventricular End-Diastolic Pressure.

**Figure S7: Automated Morphometry: Representative Image (Series III**


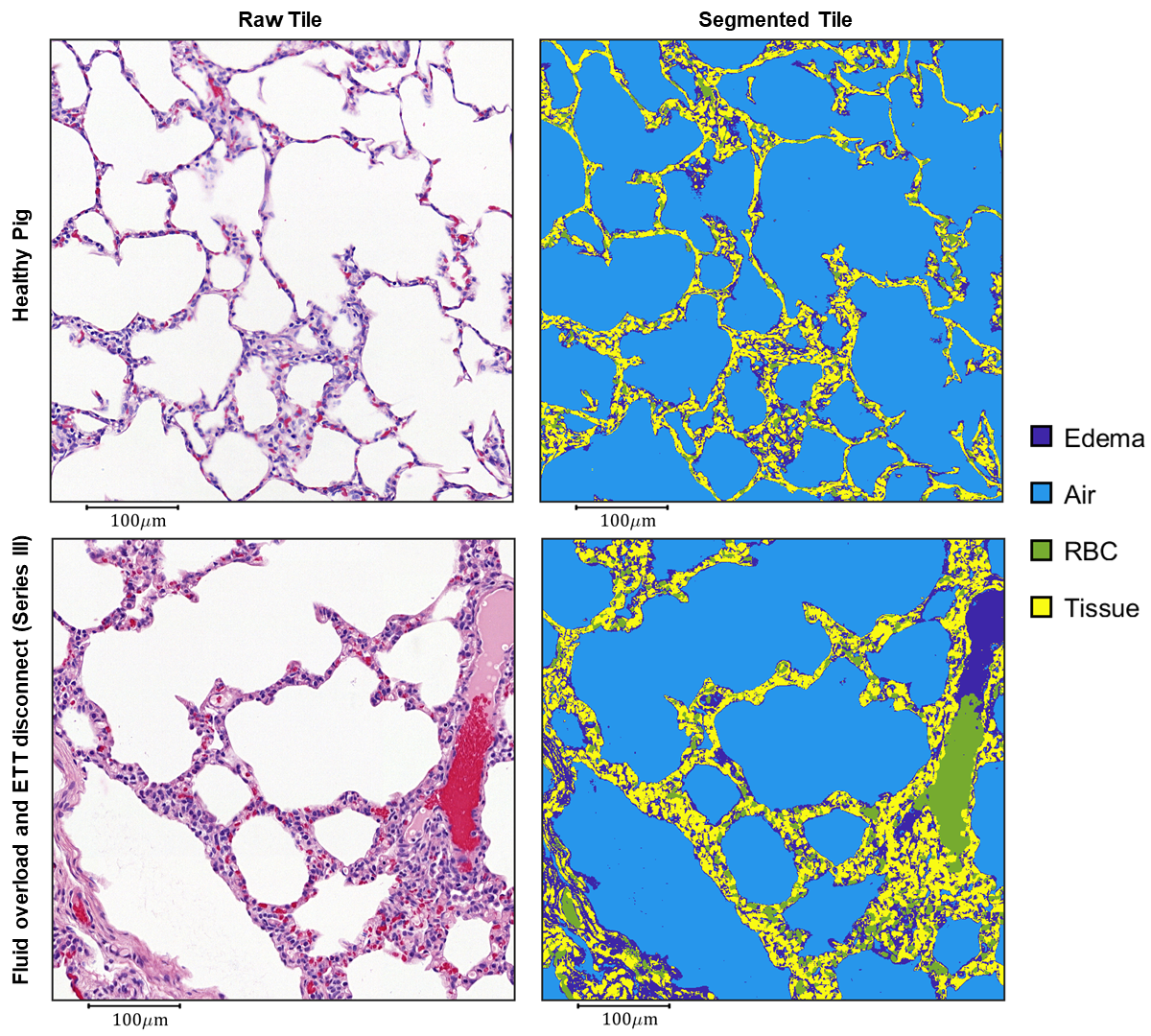


Comparison of healthy and Series III histology and automated segmentation performed via *Matlab* for morphometric analysis. Tiles from digitized slides were imported into MATLAB (MathWorks, Natick, MA) and individually converted into CIE-Lab color space. Pixels were then classified as air, tissue, edema, or RBCs using a combination of two-dimensional color-based *k*-means clustering and manual clustering seed adjustment. Scale bar is 100μm.

**TABLES - *Supplement***

**Table S1: Baseline Pulmonary and Gas Exchange Variables (Series III)**

|  | **Disconnect without Volume Loading (n=3)** | **Disconnect (n=6)** | **Control (n=6)** | **P Value** |
| --- | --- | --- | --- | --- |
| **Weight (Kg)** | 37.7 ± 4.2 | 37.0 ± 2.6 | 37.8 ± 2.1 | 0.485 |
| **Tidal Volume (mL)** | 265.0 ± 31.0 | 257.0 ± 17.0 | 265.0 ± 15.0 | 0.195 |
| **Plateau Pressure (cmH_2_O)** | 19.0 ± 0.6 | 19.0 + 0.8 | 20.0 ± 1.0 | 0.202 |
| **Driving Pressure (cmH_2_O)** | 9.0 ± 0.6 | 9.0 + 0.8 | 10.0 ± 1.0 | 0.202 |
| **C_RS_ (mL/ cmH_2_O)** | 31.0 ± 4.6 | 29.0 ± 2.2 | 27.6 ± 3.0 | 0.401 |
| **EELV (mL)** | 2432.0 ± 428.0 | 2823.0 ± 670.0 | 2788.0 ± 705.0 | 0.931 |
| **Mean FAP (mmHg)** | 71.0 ± 1.7 | 78.0 ± 12.0 | 83.3 ± 9.2 | 0.431 |
| **CVP (mmHg)** | 8.0 ± 1.5 | 8.8 ± 2.1 | 9.0 ± 2.8 | 0.911 |
| **Mean PAP (mmHg)** | 24.3 ± 4.0 | 20.0 ± 3.5 | 21.0 ± 3.4 | 0.712 |
| **PAWP (mmHg)** | 11.3 ± 1.1 | 12.2 ± 2.6 | 10.7 ± 2.8 | 0.384 |
| **LVEDP (mmHg)** | 9.0 ± 1.0 | 8.4 ± 4.3 | 7.8 ± 2.1 | 0.773 |
| **Cardiac Output (L/min)** | 5.9 ± 1.1 | 5.7 ± 0.9 | 5.1 ± 0.5 | 0.192 |
| **pH** | 7.25 ± 0.03 | 7.26 ± 0.02 | 7.25 ± 0.04 | 0.929 |
| **PaO_2_ (mmHg)** | 79.0 ± 5.7 | 77.0 ± 5.9 | 74.6 ± 1.8 | 0.288 |
| **PaCO_2_ (mmHg)** | 50.0 ± 2.6 | 56.3 ± 4.1 | 58.5 ± 7.4 | 0.526 |

In Series III, the baseline variables were measured on PEEP 10 cmH_2_O after a recruitment maneuver. Statistical comparison was performed only between Disconnect and Control groups; the pilots without volume loading are shown for comparison. ***Abbreviations*** PEEP Positive End-Expiratory Pressure; C_RS_ Compliance of the respiratory system; EELV End-Expiratory Lung Volume; FAP Femoral Arterial Pressure; PAP Pulmonary Arterial Pressure; PAWP Pulmonary Artery Wedge Pressure; LVEDP Left Ventricular End-Diastolic Pressure.

**Table S2: Cytokine Analysis (Series III)**

|  | **Disconnect (n=5)**  **Median (25% - 75%)** | **Control (n=4)**  **Median (25% - 75%)** | **P value** |
| --- | --- | --- | --- |
| **IL-1a** | 0.009 (0.003 – 0.06) | 0.006 (0.003 – 0.07) | 1.000 |
| **IL-1ra** | 0.361 (0.27 – 0.63) | 0.26 (0.15 – 0.5) | 0.413 |
| **IL- 2** | 0.10 (0.04 – 0.156) | 0.12 (0.04 – 0.13) | 0.886 |
| **IL - 6** | 0.10 (0.02 – 0.37) | 0.12 (0.04 – 0.13) | 1.000 |
| **IL - 8** | 1.67 (0.33 – 10.2) | 0.24 (0.08 – 7.1) | 0.413 |
| **IL - 12** | 0.024 (0.02 – 0.05) | 0.02 (0.01 – 0.04) | 0.556 |
| **IL - 18** | 0.3 (0.04 – 0.37) | 0.32 (0.13 – 0.4) | 0.730 |

The BAL cytokine content was similar in both groups. All results are in ng/mL

**Table S3: Histology Analysis (Series III)**

|  | **Disconnect** | **Control** | **Rank Sum Test** |
| --- | --- | --- | --- |
| ***Alveolar Edema (%)*** | 6.1 (0.7 - 42.4) | 0.0 (0.0 – 0.0) | P < 0.001 |
| ***Alveolar Edema Score (out of 4)*** | 1.0 (0.25 – 2.0) | 0.0 (0.0 – 0.0) | P < 0.001 |
| ***Interstitial Edema (%)*** | 100 (93 – 100) | 23 (10 – 82) | P < 0.001 |
| ***Interstitial Edema Score (out of 4)*** | 4.0 (4.0 – 4.0) | 1.0 (1.0 – 3.75) | P < 0.001 |
| ***Hemorrhage (%)*** | 5.2 (0 – 16) | 0.0 (0.0 – 0.0) | P < 0.001 |
| ***Hemorrhage Score (out of 4)*** | 1.0 (0.0 – 1.0) | 0.0 (0.0 – 0.0) | P < 0.001 |
| ***PMN Infiltration (%)*** | 5.9 (0.0 – 29.4) | 2.9 (0.0 – 9.3) | P = 0.045 |
| ***PMN Infiltration Score (out of 4)*** | 1.0 (0.0 – 2.0) | 1.0 (0.0 – 1.0) | P = 0.046 |

Percent of Alveoli with alveolar edema, interstitial edema, hemorrhage and intra-alveolar PMN infiltration were manually counted in total 40 slides in each group (8/animal; n = 5 animals/group). Each of the categories were scored/slide as 0 = 0%; 1 = 1 - 25%; 2 = 26 – 50 %; 3 = 51 – 75%; 4 = 76 -100%. All comparisons, using t-test were significant but since the normality and equal variance failed, all comparisons were repeated using the Mann-Whitney Rank Sum Test. The percent and score, median (25% - 75%), were higher in Disconnect compared Control in all categories but most strikingly in the interstitial edema. ***Abbreviation*** PMN polymorphonuclear.
